# Slowly walking down to the more food: relative quantity discrimination in African spurred tortoises (*Centrochelys sulcata*)

**DOI:** 10.1007/s10071-023-01812-y

**Published:** 2023-07-21

**Authors:** Masaki Tomonaga, Daiki Haraguchi, Anna Wilkinson

**Affiliations:** 1 Japan Monkey Centre, Inuyama, Aichi 484-0081 Japan; 2 University of Human Environments, Matsuyama, Ehime 790-0825 Japan; 3 Yamaguchi University, Ube, Yamaguchi 755-8505 Japan; 4 School of Life Sciences, University of Lincoln, Lincoln, LN6 7DL UK; 5 Wildlife Research Center, Kyoto University, Kyoto, 606-8203 Japan

**Keywords:** Reptile, Tortoise, Quantity discrimination, Cognition

## Abstract

Quantity discrimination, is thought to be highly adaptive as it allows an organism to select greater amounts of food or larger social groups. In contrast to mammals, the processes underlying this ability are not as well understood in reptiles. This study examined the effects of ratio and number size on relative quantity discrimination in African spurred tortoises (*Centrochelys sulcata*). To assess these effects, tortoises were presented with trays containing favored food pieces in all possible number combinations between 1 and 7. The tortoises had to approach the tray they perceived as having the larger quantity. If correct, they received one piece of food as reinforcement. The results revealed that relative quantity discrimination was influenced by the ratio between the numbers of pieces, with performance improving as the ratio between the numbers increased. This finding suggests that the approximate number system or analogue magnitude estimation may control their behavior. However, as the number size increased, their performance declined, also suggesting that the approximate number system alone could not explain the present results.

**Supplementary Information:**

The online version contains supplementary material available at 10.1007/s10071-023-01812-y.

## Introduction

Quantity discrimination is considered highly adaptive as it allows animals to choose the larger amount of food, associate with the larger group to gain access to mates, or avoid competition by choosing the smaller group (Lorenzi et al. 2021; Nieder 2020; Vallortigara et al. 2022). This area has been well-studied in mammals, birds, and fish, and their ability to discriminate depends on the ratio difference between the numbers (see Bryer et al. 2021 for a recent meta-analysis). A similar pattern of responses has been observed in amphibians (Balestrieri et al. 2019; Stancher et al. 2015; Uller et al. 2003), fish (see Messina et al. 2022 for review), and also in invertebrates (see Bortot et al. 2021; Gatto et al. 2022 for review), suggesting that this ability may originate very early in evolution.

Until recently, little was known about this ability in reptiles, a key class for developing our understanding of the evolution of cognitive abilities amongst vertebrates (Matsubara et al. 2017). A pioneering study by Miletto Petrazzini et al. (2017) showed that, whilst ruin lizards (*Podarcis sicula*) exhibited the expected patterns of discrimination of large vs. small quantities of food, there was no evidence that they could discriminate based on numerical information (Miletto Petrazzini et al. 2017). However, their performance improved when discrimination training was conducted with biologically neutral stimuli (Miletto Petrazzini et al. 2018). Several further studies with reptiles have accumulated over the past few years, especially in Testudines. There is evidence that red-footed tortoises can discriminate between stimuli based on the quantity of the reward (Soldati et al. 2017). Furthermore, Lin et al. (2021) trained Chinese stripe-necked turtles (*Mauremys sinensis*) on a discrimination task using biologically neutral stimuli and showed that they were able to discriminate up to 10 vs. 9, with performance varying with the ratio of the two numbers. Gazzola et al. (2018) also conducted an experiment on relative number judgments in Hermann's tortoises (*Testudo hermanni*) using food pieces. In their experiment, to eliminate learning factors, the tortoises were removed from the testing arena as soon as they approached one of the tables on which food items were placed and were not given reinforcement for their choice. Results of the experiment indicated that the tortoises could discriminate between 4 vs. 1 and 4 vs. 3.

**Fig. 1:**
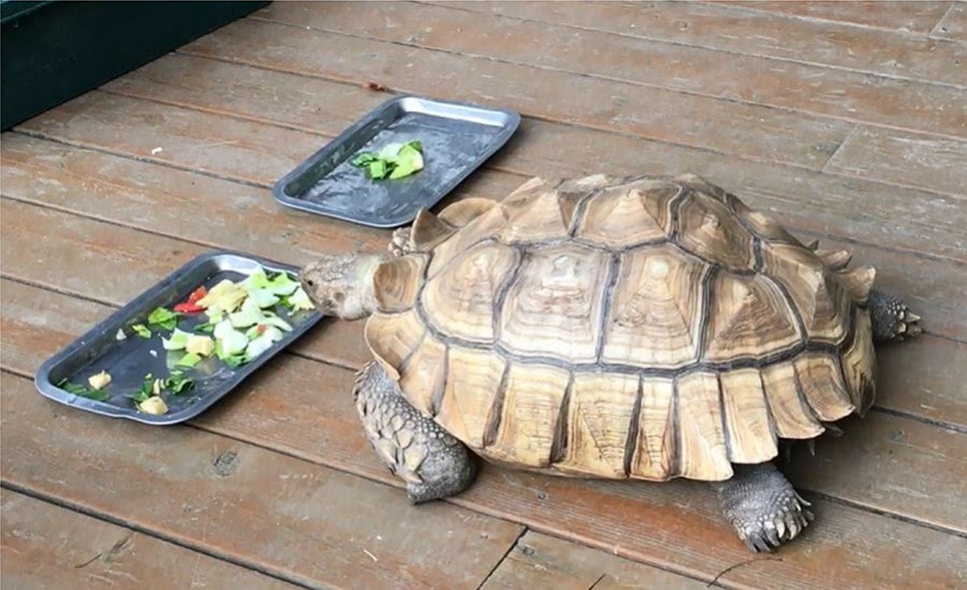
Still from an episodic video recording of African spurred tortoise (*Centrochelys sulcata*), Mike, approaching the tray with more food (see also Supplementary Video 1)

As can be seen from the studies described above, the two main types of tasks are used in studies of relative quantity discrimination in nonhuman animals (Agrillo and Bisazza 2014; Bryer et al. 2021; Gatto et al. 2022; Miletto Petrazzini et al. 2018). One is called spontaneous choice, which is a task using biologically relevant stimuli such as food (e.g., Hermann et al. 2007). According to the optimal foraging theory, animals tend to choose the one with more food when two food patches are equally available (cf. Krebs and Davies 1989; see Fig. 1 and Supplementary Video 1). The spontaneous choice task takes advantage of these habits of animals. The other is a discrimination training task using biologically neutral stimuli (e.g., dots presented on a screen) in a more controlled setting such as a laboratory (e.g., Tomonaga 2008). Although these tasks allow for more precise psychophysical measurements because they are unaffected by the rewarding properties of the stimuli to be selected, they require a great deal of training before testing. A recent meta-analysis suggests that such task differences may affect Weber ratios (Bryer et al. 2021), that is, as the ratio increases, the discrimination performance improves (Piantadosi 2016).

Human studies suggest that two systems are involved in numerical cognition (Feigenson and Spelke 2003), though there are still controversies regarding this (e.g. Starr et al. 2013). One is the *approximate number system,* or* analog magnitude estimation system*, which judges the size of a number based on the ratio of two numbers. In other words, the numerical difference between 4 vs. 2 and 20 vs. 10 is greater in the latter (2 vs. 10), but the ratio is the same (2:1). Thus, we can judge the relative numerosity of these two pairs with the same level of accuracy. This system is also limited by the Weber ratio.

The other is the *object-file system*. This system represents and tracks a relatively small number of objects individually, making it possible to track multiple objects simultaneously and accurately distinguish between different sets of quantities. However, the number of objects that can be tracked simultaneously is constrained to small numbers, at most 3–4 for human infants (Feigenson and Carey 2003). Interestingly, relative number discrimination based on the object-file system is thought not to be affected by the ratio (Feigenson and Spelke 2003). Some studies have suggested that the object-file system may exist in nonhuman animals as well as humans (Matsuzawa 1985; Murofushi 1997; Tomonaga and Matsuzawa 2002; see Agrillo et al. 2012 for a review), while other studies suggest that the results could be explained by the approximate number system alone (Rugani et al. 2011, 2013a, b, 2014; Tomonaga 2008; Tomonaga et al. 2017). Previous studies suggest that the relative number judgments in Chelonia are based on the approximate number system (Lin et al. 2021). On the other hand, if the object-file system also operates in the animals, they might be unaffected by ratios in numbers smaller than four and might show changes in performance depending not only on ratios but also on the size of the number to be compared (cf. Gazzola et al. 2018).

In the present study, we examined relative quantity discrimination using food pieces in African spurred tortoises (*Centrochelys sulcata*); this species is herbivorous and widely distributed across sub-Saharan Africa (Ritz et al. 2010). Whilst little research has been done on the visual perception of this species, research with other tortoise species has revealed relatively well-developed visual acuity (Wilkinson et al. 2013) and color perception (reviewed by Wilkinson and Glass 2018). Previous studies have also shown that tortoises have excellent visual discrimination abilities and, when given a choice, use vision to solve a task (Wilkinson and Glass 2018).

To investigate their ability to discriminate relative quantity, we conducted a mixed version of the spontaneous discrimination task in which we presented food items and differentially reinforced the tortoise's choice. Particularly, the present study examined the effects of the ratio and size of numbers on discrimination by testing all possible pairs of food pieces consisting of numbers 1 through 7. As the size of the food was not controlled for, this study assessed relative quantity information rather than focusing on numerical perception (see Methods and Discussion sections; cf. Zanon et al. 2022).

## Methods

### Participants

**Fig. 2:**
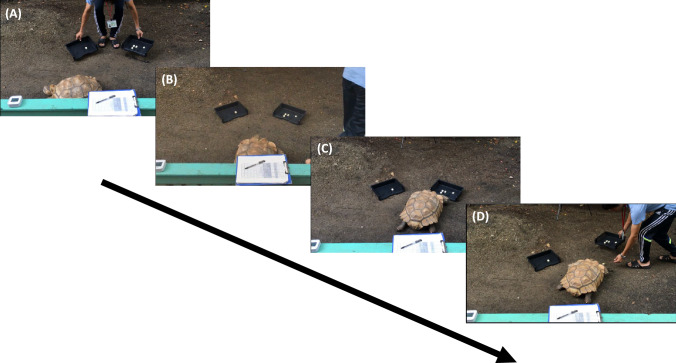
Typical trial flow of the present study. **A** Place the two trays on the left and right. **B** Place the tortoise at the starting point, facing forward as much as possible. **C** The tortoise approaching either the left or right tray. (5) Remove the tray on the approaching side and give the tortoise a piece of food if his choice is correct. See also Supplementary Video 2

Two captive-bred male African spurred tortoises (*Centrochelys sulcata*), Mike (Figs. 1 and 2; approximately 21 kg in body weight), and Rikutaro (approximately 24 kg) participated in this study.

The tortoises were part of the Kids Zoo collection at the Japanese Monkey Centre, and when not taking part in the experiment, they were housed in one of three enclosures (10 × 6 m). They were under a natural Light: Dark cycle and had access to species-appropriate food after experimentation. The tortoises were experimentally naïve at the start of the study.

### Ethics statements

The present research adhered to the Guidelines for Research Ethics of the Japan Monkey Centre, and the experimental design was approved by the Research Ethics Committee of the Centre (#2016-009). All procedures also adhered to the Guideline of the Animal Experimentation of the Japanese Society of Animal Psychology, Code of Ethics and Conduct of the Japanese Psychological Association, Ethical Guidelines for the Conduct of Research on Animals by Zoos and Aquariums of the World Association of Zoos and Aquariums (cf. Sato and Tomonaga 2010), and the Japanese Act on Welfare and Management of Animals.

### Apparatus

The present study was conducted outside in a shaded area (see Fig. 2). As the experiments were conducted outdoors, the temperature varied but was generally between 16 and 37 °C (from early August to early November 2016). Whilst this varies substantially, it is well within the normal range for this species (ClimaTemps.com 2017). A testing arena measuring 120 cm × 120 cm was marked on the ground. At one end of the arena were two presentation trays positioned 30 cm apart. Each tray consisted of a base measuring 22 cm × 30 cm and was made of matt black plastic. The trays were used to present the stimuli throughout the experiment. At the opposite end of the arena was a starting area. The front of the starting area was 75 cm from both trays. The floor of the arena was a natural gravel and mud substrate, and the tortoises could readily move around.

### Stimuli

The stimuli consisted of small pieces of preferred food (apple, cabbage, etc.). The food used varied across trials but was always the same food within a trial. The size of the pieces varied but measured approximately 2 cm × 2 cm (see Fig. 2). Each stimulus pair was made up of a different number of food pieces. The food items were randomly allocated to one of 24 (6 × 4) positions on the tray. This ensured that the density, the space occupied by the stimulus set, and the convex hull made by items varied randomly and could not be used as a cue to solve the task, although we did not manipulate the size of each food piece. All numerical combinations between 1 and 7 were tested (21 pairs total). Spatial arrangements of food items for each trial are presented in Supplementary Material 1.

### Procedure

#### General procedure

Figure 2 shows a flow of a typical trial (see also Supplementary Video 2). The tortoises were tested individually in the experimental arena. A two-alternative forced-choice procedure was used. A trial started with the tortoise being placed in the starting position, care was taken to place them centrally and not to allow their body axis to be positioned to the left or right. The tortoise was released after he had looked at (oriented his head towards) both presentation trays. Upon release, the tortoise had one minute to approach one of the presentation trays. A choice was counted if the tortoise approached within 5 cm of the tray with his head facing towards it. The time from the release to the approach was measured in seconds as latency. Only one choice was allowed. Trials were separated by an inter-trial interval of at least 30 s. The tortoises were rewarded for choosing the larger of the two stimulus sets. If the tortoise chose the larger quantity of food items, the choice was recorded, the stimuli were removed, and the tortoise was given a single piece of food. If the tortoise chose the smaller of the two quantities, his choice was recorded, the stimuli were removed, and the tortoise did not receive reinforcement. The tortoise did not get to eat the stimulus food items during the experiment. If the tortoise did not make a choice, then the animal was given a break before the trial was re-run. If the tortoise did not make a choice three times in a row, then testing was stopped for the rest of the day. The tortoises' choices were clear and unambiguous as the stimulus trays were presented 30 cm apart. The experimenter was positioned so that they were out of view of the tortoise for the duration of the trial.

For each trial, the tortoise was presented with a different number of food items on each presentation tray. The side of the presentation was pseudorandomized across trials so that the larger stimuli were never presented on the same side more than three times in a row. Scent cues were controlled for by rubbing both trays with chopped food before the onset of the trial. This ensured that smell of the food was equal on both presentation trays.

#### Preliminary training

Prior to the main experiment, the tortoises underwent preliminary training. Preliminary training began with 7 vs. 0 and followed by 7 vs. 1 until their performance became stable. A session consisted of 6 trials. Mike received 3 sessions for 7 vs. 0 and 5 sessions for 7 vs. 1. Rikutaro received 3 sessions for 7 vs. 0 and 3 sessions for 7 vs. 1. Accuracy of 7 vs. 1 was 87% for Mike and 89% for Rikutaro, respectively.

#### Main experiment

After the preliminary training, we conducted the main experiment. All 21 pairs of combinations of numbers from 1 to 7 were presented in a random order. The tortoises received each stimulus pair in blocks of six trials (that made up one session). Tortoises received six sessions (36 trials) for each pairing. 1 to 4 experimental sessions were conducted per day, up to 5 days per week.

#### Data analysis

General linear mixed modeling (GLMM) was used to analyze the data. For accuracy, the objective variable (response variable) was the proportion of correct choices (binomial distribution), and three types of fixed effects were introduced. The first was the Ratio difference between the two numbers. In this study, the ratio was defined as the difference between the larger and smaller number divided by the smaller number [(Large–Small)/Small]. The second was the Larger of the two numbers, as a measure of the size of the numbers presented. These explanatory variables were log-transformed to ordinary logarithms. We further included Temperature as the third fixed effect since, as mentioned earlier, there were relatively large temperature variations during the experiment. In addition, Participants and Sessions (nested in Participants) were used as random effects. Among the random-intercept models combining these effects and all interactions, the model with the smallest Akaike Information Criterion (AIC) was selected. In addition, each parameter estimate was evaluated based on 95% confidence intervals using Wald's test statistic. The latency (lognormal distribution) was also analyzed with GLMM. For further reference, we also separately performed GLMM analyses for each individual since the experiment was conducted with two tortoises. Raw data for each individual used for data analyses are shown in Supplementary Material 2.

## Results

**Fig. 3:**
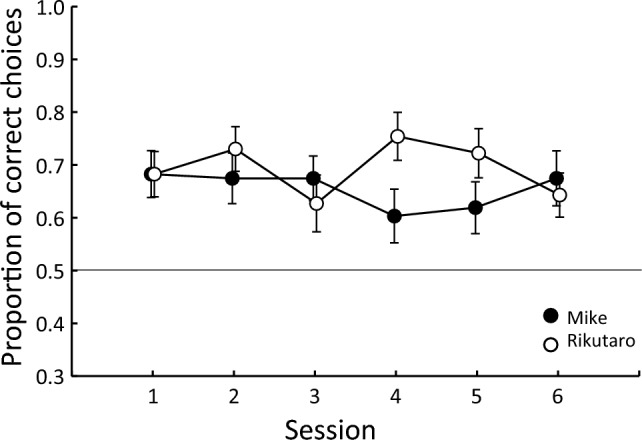
Mean proportion of correct choices for all pairs as a function of sessions. Error bar shows standard error of the mean across pairs

Figure 3 shows the mean proportion of correct choices for all pairs by session to examine the learning effect during the experiment. Clearly, the tortoises showed above-chance performances, and there was no significant learning effect during the experiment, suggesting that they readily processed the information.

**Fig. 4:**
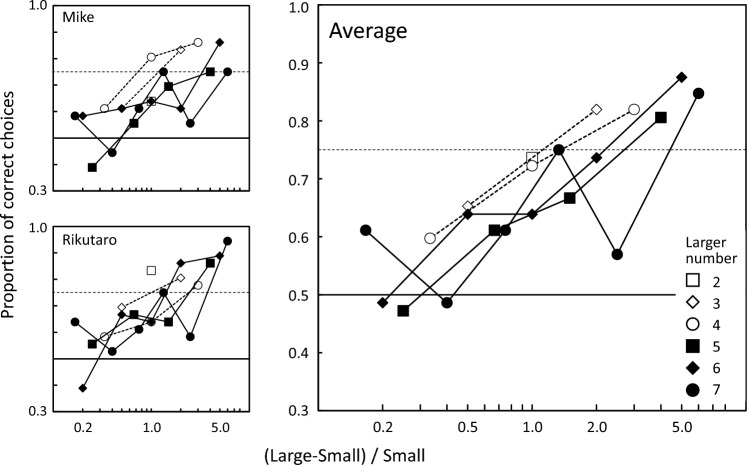
Proportion of correct choices for each pair. The horizontal axis shows the ratio in the form of difference between the larger and smaller number divided by the smaller number on a logarithmic scale. The larger number is indicated by different symbols. The results for each tortoise are shown on the left, and the average of the two is shown on the right

Based on GLMM, a model including Ratio, Larger number, and their interaction was selected. Temperature was not included in the selected model. Table 1 shows a summary of the statistical analyses. Figure 4 shows the proportion of correct choices for each pair. The horizontal axis is the ratio, and the larger number is indicated by different symbols. The accuracy of the relative quantity discrimination by the tortoises increased as the ratio increased. In addition, their performances worsened as the larger number increased. However, this effect varied across individuals: Rikutaro did not show a significant effect of the larger number.

**Table 1:** Summary of generalized linear mixed model analyses

Tortoise	AIC	Likelihood	Parameter					Wald			
			Fixed effects	Estimate	SE	95% CI		Statistics	*p*	Random effect	SD
Accuracy											
Mike	395.8	− 192.9	Intercept	1.348	0.406	0.553	2.143	3.322	0.0009	Session	1.8E− 08
			log[(L−S)/S]	3.681	1.409	0.920	6.442	2.613	0.0090		
			log(Larger number)	− 0.957	0.557	− 2.049	0.134	− 1.719	0.0856		
			Interaction	− 3.690	1.837	− 7.290	− 0.089	− 2.008	0.0446		
Rikutaro	374.3	− 183.2	Intercept	1.693	0.523	0.668	2.718	3.237	0.0012	Session	0.119
			log[(L−S)/S]	1.027	0.210	0.615	1.438	4.887	< 0.001		
			log(Larger number)	− 0.939	0.583	− 2.081	0.203	− 1.611	0.1072		
Average	769.8	− 378.9	Intercept	1.439	0.294	0.862	2.015	4.893	< 0.001	Session:partcipant	0.071
			log[(L−S)/S]	2.587	1.011	0.605	4.568	2.559	0.0105	Partcipant	0.035
			log(Larger number)	− 0.952	0.402	− 1.740	− 0.165	− 2.370	0.0178		
			Interaction	− 2.076	1.321	− 4.665	0.513	− 1.571	0.1161		

Tortoise	AIC	Likelihood	Parameter									
			Fixed effects	Estimate	SE	95%CI		df	*t* statistic	*p*	Random effect	SD
Latency												
Mike	1203.1	− 598.6	Intercept	1.993	0.033	1.929	2.057	125	61.080	< 0.001	Session	0.309
Rikutaro	1004	− 499	Intercept	1.899	0.029	1.843	1.956	125	65.830	< 0.001	Session	0.275
Average	2214.3	− 1103.2	Intercept	1.946	0.047	1.854	2.038	1	41.510	0.0153	Session:Partcipant	0.293
											Partcipant	0.059

*L* larger number of the pair, *S* smaller number

**Fig. 5:**
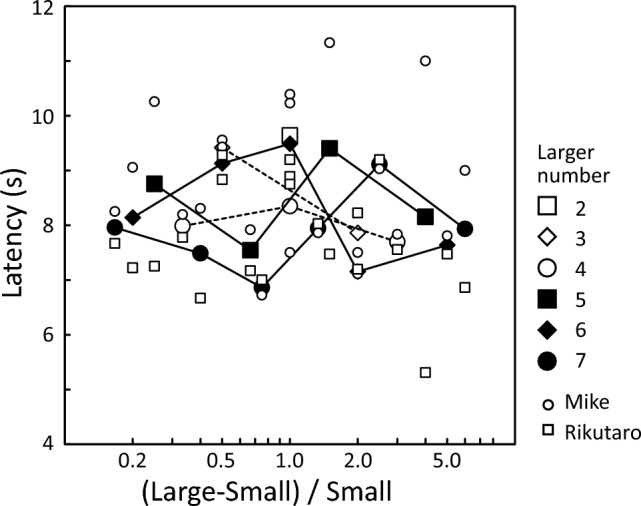
Mean latency for each pair. The larger number is indicated by different symbols. Individual data are also plotted in this graph with smaller symbols

Figure 5 shows the mean latency for each pair. The mean latency for Mike was 8.8 s, and 7.8 s for Rikutaro. Latency was neither affected by the ratio, the larger number, nor the temperature; the null model was also selected based on GLMM (Table 1).

**Fig. 6:**
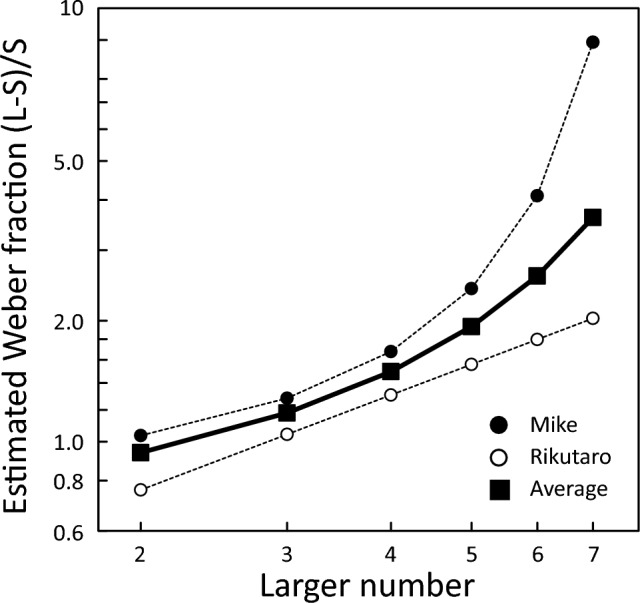
The Weber fraction for each larger number. Individual results are also shown. The Weber fraction was defined as (Large–Small)/Small at 75% correct choice rate

Based on the selected models, the ratio of numbers when the correct choice rate reached 75%, or *Weber fraction*, was calculated. However, since the larger number was incorporated into the model using the overall data and the model conducted for each individual, the Weber fraction was calculated for each larger number. The results are shown in Fig. 6. Weber fraction was not constant across the size of the numbers presented, and as the larger number increased, Weber fraction also increased.

**Fig. 7:**
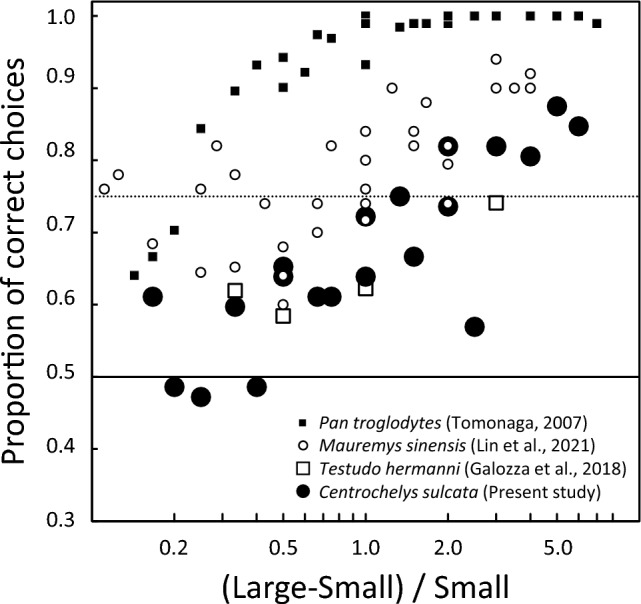
Comparison of the present results with the previous studies. The results of the present study and those of the two turtle species are plotted. In addition, the results of the chimpanzees are also plotted for reference

We compared the present results with those of previous studies with tortoises (Fig. 7), Hermann's tortoises (Gazzola et al. 2018), and Chinese stripe-necked turtles (Lin et al. 2021). Note that the former was conducted with spontaneous choice, and the latter was conducted with discrimination training. Both African spurred tortoises and Hermann's tortoises belong to the family Testudinidae, and the Chinese stripe-necked turtle belongs to the family Geoemydidae, but all three species belong to the superfamily Testudinoidea. Also shown for reference are the results for chimpanzees (Tomonaga 2008), which were conducted in a discrimination training task.

Figure 7 shows that the results for the two Testudinidae species were relatively similar, whereas the results for the Chinese stripe-necked turtles were better than those for the other two species. In contrast, the chimpanzees' performances were better than those of the tortoises (cf. Bryer et al. 2021).

## Discussion

In this study, we conducted a relative quantity discrimination task on captive African spurred tortoises using food pieces as stimuli. The results revealed that the relative quantity discrimination by the tortoises was influenced by the ratio between the numbers being compared: as the ratio increased, the proportion of correct choices also increased. These results indicate that their discrimination was governed by the approximate number system or analogue magnitude representations. On the other hand, the opposite tendency was observed for the larger numbers, a measure of number size: as the larger number increased, their performance tended to decrease. Therefore, the Weber fraction defined by the 75% accuracy also tended to increase as the larger number increased. If the approximate number system alone is involved in processing then performance should only be affected by the ratio, regardless of the size of the number. The present results, in which performances changed with the number size, might suggest that the object-file system is also involved in the relative quantity discrimination in tortoises. However, if the object-file system is involved in the relative discrimination of small numbers, then accuracy should be constant regardless of the ratio (Feigenson and Spelke 2003; Agrillo et al. 2012). Nevertheless, the increasing trend as a function of the ratio was kept even when the larger number was less than or equal to 4 (see open symbols in Fig. 3). Thus, a simple object-file system model would not be enough to explain the present results. To further address this issue, tests using smaller numbers might be helpful (cf. Tomonaga 2008).

One possible reason for the decrease in accuracy as the number increased might be that the present task used food items as stimuli. The tortoises might not care to choose either tray if the incentive (reinforcement value) of "a lot of food" in front of them was high, for example, 7 vs. 6. Even if they recognized the difference. One would also have to consider that if the object to be compared is a primary reinforcer, such as food, they may not be comparing differences in the number of pieces but in the "reward value". This might be one of the reasons for the lower performances in spontaneous choice tasks, although as the animals were reinforced with a single piece of food after a correct choice, this is less likely to impact our experiment. In chimpanzees, for example, the number size effect was not found in a PC-controlled relative number judgment task (Tomonaga 2008). In addition, they showed lower performances for the spontaneous choice task with food items similar to the present experiment than for the computer-controlled discrimination task (Tomonaga and Mori 2012). Future studies with much larger amounts of food may help address this issue.

In the study using neutral objects by Lin et al. (2021), the Chinese stripe-necked turtles showed higher accuracy than African spurred tortoises and Hermann's tortoises (Gazzola et al. 2018). These results might not reflect a species difference but rather procedural differences; Lin et al. used the discrimination training task with neutral objects, but Gazzola et al. and our experiment used food items. The number size effect was also observed in the Lin et al. study. Interestingly, however, our reanalysis showed the opposite tendency to our results: performances improved with increasing the larger number. This discrepancy may be an outcome of the training effect that their experiment successively progressed, with better performances in the later phases using larger numbers.

In the present experiment, we systematically investigated the relative quantity or number discrimination with a wide range of numbers (1–7) in the African spurred tortoises. Both individuals in this experiment showed a ratio effect, but the effect of the larger number was not observed in one of the tortoises, Rikutaro. To verify the generality of the present results, more individuals would be needed. Studies with individuals in different facilities and field experiments in the wild may also be fruitful in strengthening our conclusion.

The present study could not rule out the possibility that other physical cues such as area size, length of circumference etc. (cf. Tomonaga 2008; Tomonaga et al. 2017; Zanon et al. 2022) were used by the tortoises. It has been reported that tortoises can discriminate different quantities. Soldati et al. (2017) trained red-footed tortoises (*Chelonoidis carbonaria*) to associate a colored stimulus with a specific amount of reward that was either large or small (125 mm^3^ vs. 27 mm^3^). The tortoises readily learned this discrimination and were willing to work harder (walk further) for the stimulus associated with the larger reward amount. Gazzola et al. (2018) examined not only relative discrimination of the number of food items but also the size of a single food. They found that size discrimination was also affected by the ratio but pointed out that different mechanisms may be involved in each discrimination. Considering these results, we cannot completely exclude the possibility that cues other than number may have impacted the tortoises' behavior. Further experiments controlling for these physical variables will be needed in the future. An approach which uses artificial stimuli would also allow counterbalancing of the positive stimulus which, while is not necessary to assess numerical discrimination, would allow interesting comparisons of rates of learning between those rewarded for selecting the higher numerosity and those rewarded for selecting the lower one (e.g., Kilian et al. 2003; Potrich et al. 2022). It would also be valuable to examine how tortoises perform relative discrimination of these physical variables under conditions where numerical variables do not serve as discriminative cues (cf. Lorenzi et al. 2021).

The present experiment suggests that the relative quantity discrimination by the tortoises is influenced not only by the ratio but also by the number of objects. This initial work paves the way for future work investigating this phenomenon in further detail, such as working with more animals to assess how representative these findings are of the species in general and further probing which aspects of the stimuli the animals are responding to by using neutral stimuli such as those used by Tomonaga (2008).

## Supplementary Information

Below is the link to the electronic supplementary material.

Supplementary file1 African spurred tortoise, Mike, approaching the tray with more foods (MP4 26196 KB)

Supplementary file2 A tortoise approaching the tray with four food pieces (MP4 22881 KB)

Supplementary file3 Raw datasheets for the present study. Data are arranged in the order of date. Information for spatial configuration of food items are also provided (XLSX 115 KB)

Supplementary file4 Data set for statistical analyses (XLSX 203 KB)

## Data Availability

All data used in this study are available from the supplementary materials.
